# Orthostatic *Ex-Vivo* Lung Perfusion (EVLP): A Proof of Concept

**DOI:** 10.3389/ti.2024.13178

**Published:** 2024-07-31

**Authors:** Massimo Boffini, Andrea Costamagna, Matteo Marro, Erika Simonato, Paola Cassoni, Luca Bertero, Vito Fanelli, Cristina Barbero, Luca Brazzi, Mauro Rinaldi

**Affiliations:** ^1^ Cardiac Surgery Division, Surgical Sciences Department, Citta della Salute e della Scienza, University of Torino, Turin, Italy; ^2^ Anesthesiology and Intensive Care Division, Surgical Sciences Department, Citta della Salute e della Scienza, University of Torino, Turin, Italy; ^3^ Pathology Unit, Medical Sciences Department, Citta della Salute e della Scienza, University of Torino, Turin, Italy

**Keywords:** lung transplant, animal model, EVLP, organ preservation, perfusion

## Abstract

The key goal in lung donation remains the improvement of graft preservation with the ultimate objective of increasing the number and quality of lung transplants (LTx). Therefore, in recent years the field of graft preservation focused on improving outcomes related to solid organ regeneration and restoration. In this contest Ex-Vivo Lung Perfusion (EVLP) plays a crucial role with the purpose to increase the donor pool availability transforming marginal and/or declined donor lungs suitable for transplantation. Aim of this proof of concept is to test the safety, suitability and feasibility of a new tilting dome for EVLP designed considering the dorsal lung areas as the “Achilles’ heel” of the EVLP due to a more fluid accumulation than in the supine standard position.

## Graphical Abstract

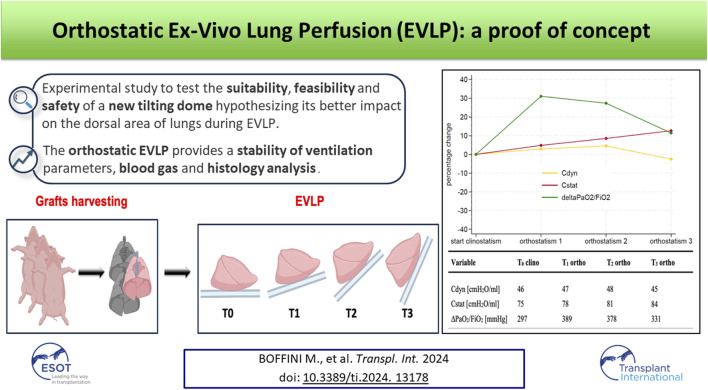

## Introduction

Although nowadays lung transplantation (LTx) is a well-established treatment for patients with end-stage lung diseases, shortage of suitable lung grafts is still a major limitation for an extensive application of this therapy and the low number of acceptable grafts negatively impacts waitlist mortality [1]. However, increasing the quantity of grafts by accepting donor organs of inferior quality might increase the incidence of primary graft dysfunction and worsen recipients’ outcomes [2]. *Ex Vivo* Lung Perfusion (EVLP) has emerged as an effective technique for preserving, evaluating and eventually reconditioning donor lungs [2–7]. Thus lung transplant activity may be increased by 15%–30% in Lung Transplant Programs adopting EVLP protocols [8–10].

In the last years we faced an increase in clinical experience and an implementation of this technology, configurating EVLP as a tool providing an opportunity to treat initially rejected donor lungs before lung transplantation: antimicrobial therapy [11, 12], interleukin gene therapy [13], cytokines absorption [14] are some examples of potential therapeutical applications. Despite this wider application, there are still remaining issues concerning EVLP use and one of these might be the supine position of the graft during the procedure. This can result in hyper-perfusion of the lower-dependent regions and hypo-perfusion of the nondependent regions according to the West three-zone model [15]. The subsequent asymmetric ventilation-perfusion distribution might increase capillary shear stress resulting in increased vascular permeability with pulmonary edema accumulation [16]. Thus Niikawa et al. [17], in a porcine model study comparing prone and supine positioning of lungs during EVLP, showed less ischemia-reperfusion injury and improved lung function in the prone group. The same authors confirmed this results in human lungs rejected from clinical use, showing in addition a selective improvement in terms of lung function and cytokine load in the lower lobes of the proned lungs [18]. In the same way Ordies et al. [19] highlighted a more concentrated fluid accumulation in lower-dependent regions in the prone group of porcine lungs mounted on a normothermic EVLP compared to the supine group, despite oxygenation (P/F), dynamic compliance and peak airway pressures were comparable between both groups. To the best of our knowledge, only two cases of successful lung transplantation after normothermic acellular EVLP with prone positing, indicated by the clinical evidence of lung oedema, have been published so far [20].

Although promising, prone positioning of the EVLP lungs raises some technical concerns about feasibility and safety in the routinary clinical practice. First, pulmonary arterial and left atrial cuffs and cannulas are non-accessible during prone position. Second, the anterior face of the grafts might be compressed or injured during lung manipulation and positioning. Third, the anterior regions might in turn experience hyper-perfusion during the procedure. The current evidence focuses on the comparison between supine and prone position, but theoretically, the best option would be the possibility of switching between supine and prone position (and vice-versa) during the perfusion. Although potentially beneficial in terms of ventilation/perfusion mismatch, this approach could raise some concerns related to the risk of repeated graft manipulation, break in sterility, accidental mispositioning of vascular cannulas and endotracheal tube and eventually lung lesions.

Considering the dorsal lung areas as the “Achilles’ heel” of the EVLP, we experimented a self-designed tilting organ dome allowing a graft positioning change from 0° to approximately 90° during the procedure, miming the physiological position of human lungs in a standing subject. This would allow to overcome the aforementioned concerns about sterility break and technical issues. Aim of this experimental study was to test the feasibility and safety of the new dome hypothesizing its better impact on the dorsal extravascular fluid accumulation observed during EVLP.

## Methods and Materials

### Study Design

Three adult pigs (around 200 kg each) of a slaughterhouse were included in the study. All animals were treated according to the European Commission guidelines about animal care during butchery [21]. After euthanasia, the heart-lung blocks were procured by the veterinary surgeon in a standard fashion and then anterogradely flushed with cold (4°C) Perfadex^®^ solution (70 mL/kg). Only one of the three blocks presented with no injuries due to the retrieval and/or macroscopic signs of pulmonary damage thus it was considered feasible for the experiment. One was excluded for macroscopical edema from the trachea and one for pleuro-parenchymal lesions with significative air leak. A second retrograde cold flush of 1,000 mL of the same solution was then performed in the block and then subjected to 4 h of cold static storage, mimicking the setting of donation after circulatory death (DCD). Differently from other study protocols [17, 19], due to the slaughterhouse setting and rules, the animals were not intubated prior death and grafts were harvested without modification of the butchery production line due to slaughterhouse regulation and according to the animal welfare.

### *Ex Vivo* Lung Perfusion

**FIGURE 1:**
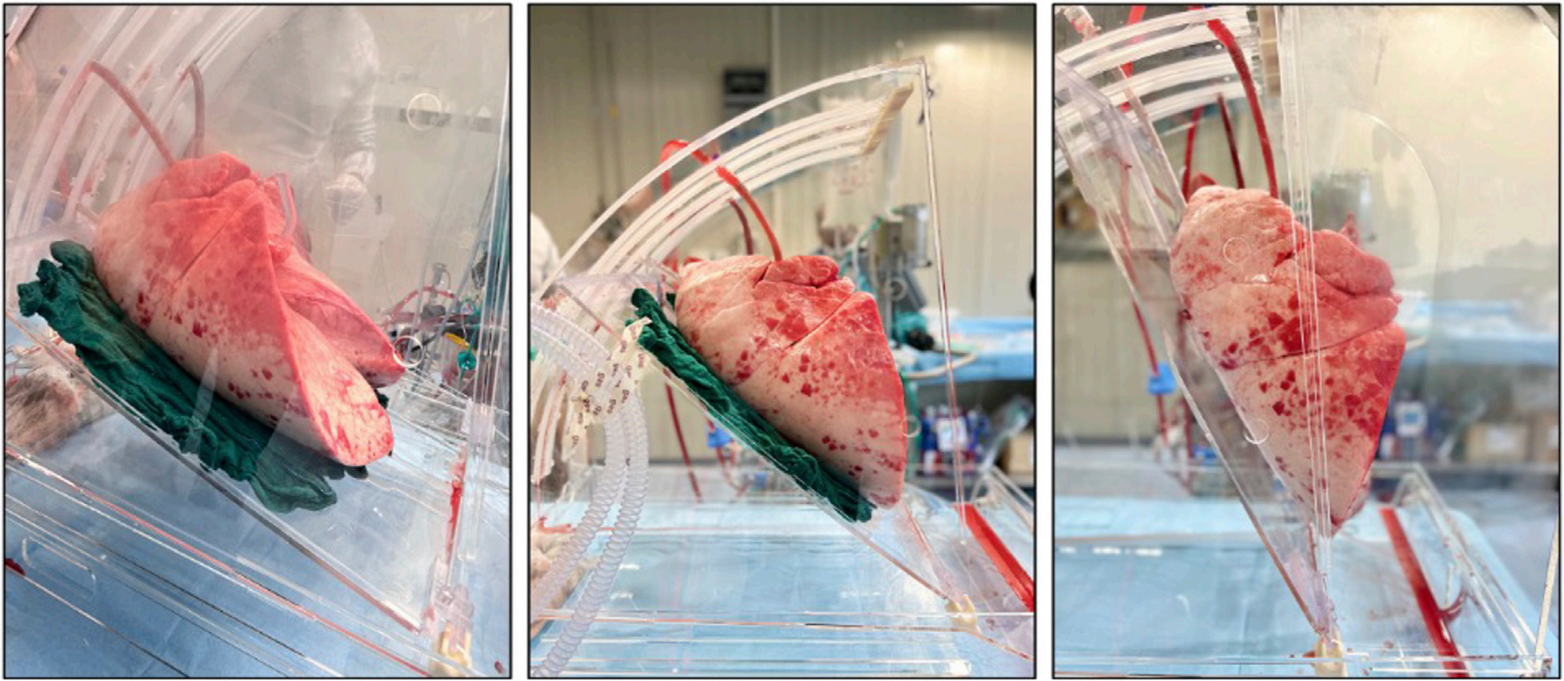
Lung block positioned in the dome with different degree of inclination.

Once in the dedicated facility, the left atrial (LA) cuff was trimmed and sewn to a dedicated cannula with a 4-0 polypropylene running suture and the pulmonary artery (PA) cannula simply inserted proximally to the artery bifurcation and secured with two heavy silk ties. A conventional endotracheal tube was then inserted and secured. The components of the circuit were: a centrifugal pump, a leukocyte filter, a hollow-fiber oxygenator, a heat exchanger and a hard-shell reservoir. The circuit was primed with 2.0 L of acellular Steen solution. 500 mg methylprednisolone, 3000 IU of unfractionated heparin were also added to the solution of perfusion. The new dome (PerLungs^®^, Aferetica s.r.l., Bologna, Italy) (Figure 1) is a novel chamber with a mobile base manually tilting from 0° to approximately 90° on the coronal plane. A hook in the proximal part of the tilting plate serves to anchor the lungs at the carina and it keeps the graft suspended when in orthostatic position. The hook can be placed at different lengths according to the dimensions of the graft. On the side of the dome, there are working ports to allow the crossing of additional catheters used during perfusion. The anterior face of the chamber presents three slits the circuit can pass through. The lungs were thus taken to the EVLP dome and connected to the circuit: the PA cannula was then connected to the circuit and anterograde flow was started. The LA cannula was then deaired and connected to the circuit. According to our protocol, the target perfusion flow consists of 40% of the donor predicted cardiac output. Following the principles of gradual rewarming and stepwise increase of vascular flow, the perfusion is then initiated with subsequent increase of the calculated target flow to reach the target cardiac output in 1 hour. At 10 min, the flow is increased to 20% and the temperature is set to 30°C. The flow is progressively increased to 30%, 50%, 80% and finally 100% of target, respectively every 10 min. The temperature of the heat exchanger is set to 37°C after 20 min from the beginning of perfusion and ventilation is initiated (FALCO 202 EVO, Siare Engineering International Group Srl, Italy) with a standard ventilation (Vt 7 mL/kg, PEEP 5 cm H_2_O and 7 cycles/min, FiO_2_ 21%.) when the temperature reaches 32°C. Just before the lungs are ventilated, the oxygenator is connected to a gas mixture (86% N2, 8% CO2 and 6% O2) tank. The sweep gas is set to achieve a pCO2 between 35-40 mmHg. Lastly, the left atrial pressure is carefully maintained to a stable value of 3–5 mmHg by adjusting the venous return to the reservoir. The lungs reached the steady state and they were subsequently perfused for up to 4 h. The tilting plate is progressively moved to reach a nearly orthostatic position during the first hour of perfusion and the graft is kept in the orthostatic positing till the end of perfusion.

### Lung Function Assessment on EVLP

**FIGURE 2:**
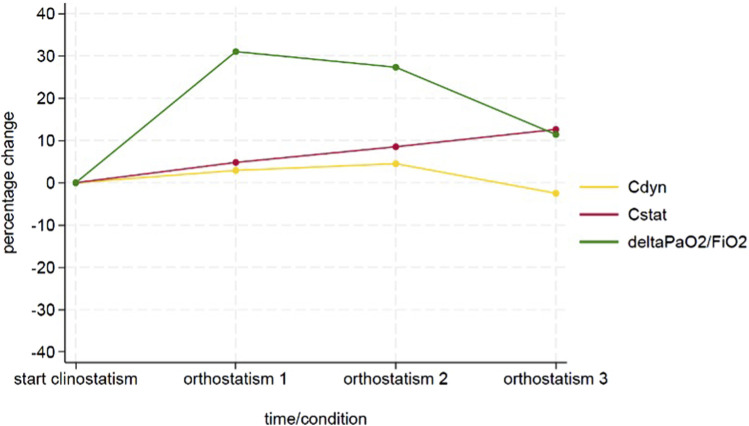
Time course of the delta PaO2/FiO2, Cdyn and Cstat variables.

After the beginning of ventilation the dome base was positioned from 0°C to approximately 90°C and lungs were derived hourly with physiologic parameters (Vt 10 mL/kg, PEEP 5 cm H2O and 10 cycles/min, FiO2 100% - Figure 2). The following measurements were performed:

• dynamic compliance (cdyn) that was calculated according to the following formula:

$$\text{Cdyn}=\text{Vt}/\left(\text{PiP}-\text{PEEP}\right)\;\left[\text{mL}/{\text{cmH}}_{2}O\right]$$

where PiP was peak inspiratory pressure.

• static compliance (cdyn) that was calculated according to the following formula:

$$\text{Cstat}=\text{Vt}/\left(\text{Pplat}-\text{PEEP}\right)\;\left[\text{mL}/{\text{cmH}}_{2}O\right]$$

where Pplat was plateau inspiratory pressure, calculated during an inspiratory hold maneuver.

• assessment of gas exchange: blood gas tests were performed on perfusate samples to calculate the difference between left atrial PaO2 and pulmonary artery PaO2 with an Oxygen concentration on the ventilator of 100% for 5 min

$$\text{delta PaO}2/\text{FiO}2={\text{PaO}}_{2}/{\text{FiO}}_{2\text{LA}}-\;{\text{PaO}}_{2}/{\text{FiO}}_{2\text{PA}}\;\left[\text{mmHg}\right]$$

**FIGURE 3:**
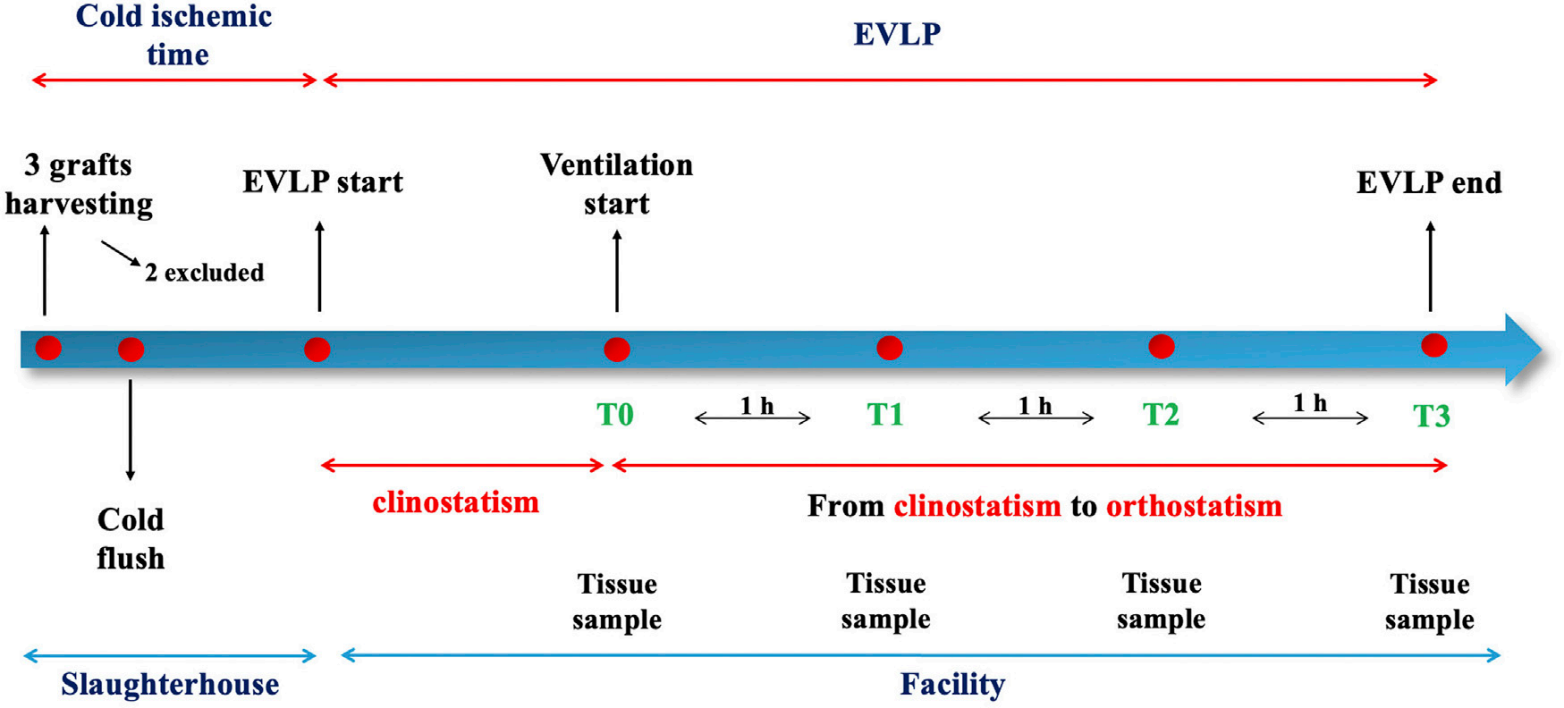
Timeline of the procedure.

All the variables were reported as absolute values and percentage change between the value measured at each timepoint during orthostatism and its baseline measured in supine position. Figure 3 represents the study timeline.

### Lung Tissue Sample Collection

Lung tissue samples were collected from the lower left lobe at the beginning of the procedure (supine position) and every hour of EVLP (in the orthostatic position), then fixated in formaldehyde 6%, embedded in paraffin, sliced (4 mm) and stained with hematoxylin and eosin. A lung pathologist studied each biopsy for presence of congestion, alveolar edema, alveolar granulocyte infiltration, interstitial granulocyte infiltration, alveolar microhemorrhage and micro-thrombosis. Additional histological analysis were performed on the entire block at the end of the procedure, one for each of the six lobes.

## Results

During the entire procedure, lungs maintained a stable gross aspect with no evident lobar congestion and/or atelectasis. Physiological ventilation parameters and blood gas analysis variation during EVLP are shown in Figure 2. Lungs maintained satisfying gas exchange parameters throughout the experimental time: the ΔPaO2 (PaO2 pre - PaO2 post) was >300 mmHg for 4 h, with a mean value of 366 ± 30 mmHg. Peak and plateau airway maintained a stable value during the procedure; similarly dynamic and static compliance did not worse significantly over time (Table 1). A representative histology sample of each region at the end of the perfusion is presented in Figure 4.1. The hematoxylin and eosin stain analysis showed neither edema nor congestion in all the samples from the first to the fourth hour of the procedure (Figure 4.2), with a minimum focal alveolar septal thickening; the granulocytic infiltration was sporadic in the lung interstitium and absent in the alveolus. Moreover, neither hemorrhage nor micro-thrombosis have been detected.

**TABLE 1:** Time course of physiologic and respiratory mechanic variables during EVLP.

Variable	T_0_ clino	T_1_ ortho	T_2_ ortho	T_3_ ortho
Cdyn [cmH_2_O/ml]	46	47	48	45
Cstat [cmH_2_O/ml]	75	78	81	84
ΔPaO_2_/FiO_2_ [mmHg]	297	389	378	331

List of abbreviations: Cdyn dynamic compliance; Cstat static compliance; ΔPaO_2_/FiO_2_ difference between left atrial PaO2 and pulmonary artery PaO2 with an 1.0 oxygen concentration.

**FIGURE 4:**
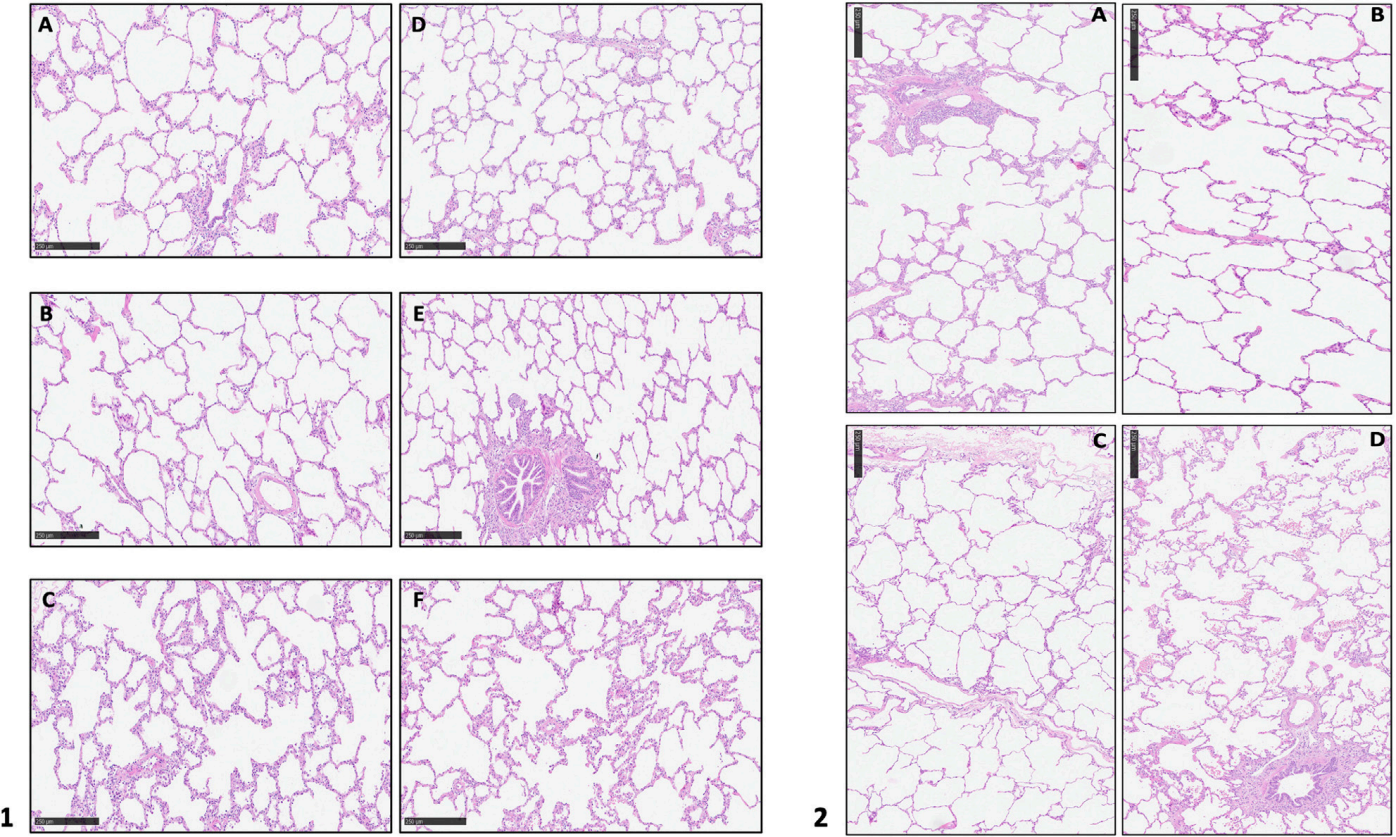
**1**. Hematoxylin and eosin stain analysis of the whole block at the end of the perfusion. **(A)**: upper right lobe; **(B)**: medium right lobe; **(C)**: lower right lobe; **(D)**: upper left lobe; **(E)**: accessory left lobe; **(F)**: lower left lobe; **2**. Hematoxylin and eosin stain analysis at different time of perfusion: **(A)**: T0; **(B)**: T1; **(C)**: T2; **(D)**: T3.

## Discussion

The key goal in lung donation remains the improvement of graft preservation with the ultimate objective of increasing the number and quality of transplants. Therefore, in recent years the field of graft preservation focused on improving outcomes related to solid organ regeneration and restoration. In this contest EVLP plays a crucial role with the purpose to increase the donor pool availability transforming marginal and/or declined donor lungs suitable for transplantation. Aim of this proof of concept is to test the safety, suitability and feasibility of a new tilting dome for EVLP designed considering the dorsal lung areas as the “Achilles’ heel” of the EVLP due to a more fluid accumulation and a ventilation-perfusion mismatch in the supine standard position.

Mechanical ventilation in the prone position, first reported in 1970 [22], has been evaluated as a strategy to enhance oxygenation and lung recruitment in acute respiratory failure. The mechanisms by which no-supine positioning may benefit patients with acute respiratory distress syndrome (ARDS) undergoing mechanical ventilation include increasing end-expiratory lung volume, improving ventilation–perfusion matching and preventing ventilator-induced lung injury by more uniform distribution of tidal volume through lung recruitment and alterations in chest wall mechanics [23]. Based on this postulate, the porcine model study of Niikawa et al. [17] demonstrated that prone positioning during EVLP was significantly associated with less lung weight gain, better P/F ratio and improved compliance, lower inflammatory cytokines, and lower ALI grade difference than supine positioning in the control group. Some months later Ordies et al. [19] demonstrated that prone positioning of the donor porcine graft during EVLP is feasible and resulted in more homogenous distribution of interstitial fluid.

In our experience, the positioning of graft at almost 90° provided a stability of ventilation parameters and blood gas analysis data. The macroscopic evaluation (Video 1) also demonstrated a better expansion of the dorsal graft areas, miming the physiological movement of the lungs of a standing subject.

This proof-of-concept experiment has several limitations related with the experimental setting and the limited number of tests. The animal model mimics DCD donation with some differences mainly related with the suspension of mechanical ventilation and a delayed flush of preservation solution. The paper describes a single experiment without a control group of standard EVLP. This does not allow to speculate about a potential positive and meliorative effect of the orthostatic position during the ex-vivo perfusion. The paper wants to be only a “proof of concept” regarding the feasibility of tilting the graft during EVLP. A direct comparison with a control group may add specific insights regarding its efficacy and/or potential improvement and it may allow a deeper evaluation exploring if the tilting dome only moves the fluid according to the gravity law or a more “physiological” position might act through more complex pathways. Moreover, the absence of a portable x-ray machine did not allow a radiological evaluation of the graft in terms of lobar congestion. However, our initial test might suggest that this approach is feasible without compromising the usual stability obtained maintaining the graft in the standard position.

## Conclusion

Further experimental tests are mandatory to better analyze the safety, feasibility and potential benefits of a “stand-up position” of the graft on the physiological respiratory mechanics and blood gas analysis data.

## Data Availability

The original contributions presented in the study are included in the article/Supplementary Material, further inquiries can be directed to the corresponding author.
